# Nephroprotective effects of diminazene on doxorubicin-induced acute kidney injury in rats

**DOI:** 10.1016/j.toxrep.2023.11.005

**Published:** 2023-11-10

**Authors:** Yousuf Al Suleimani, Raya Al Maskari, Badreldin H. Ali, Haytham Ali, Priyadarsini Manoj, Ali Al-Khamiyasi, Aly M. Abdelrahman

**Affiliations:** a Department of Pharmacology and Clinical Pharmacy, College of Medicine and Health Sciences, Sultan Qaboos University, P.O. Box 35, Al Khod 123, Oman; b Department of Animal and Veterinary Sciences, College of Agricultural and Marine Sciences, Sultan Qaboos University, Muscat 123, Oman

**Keywords:** Doxorubicin, Diminazene, ACE2 activator, Lisinopril, Valsartan, Acute kidney injury

## Abstract

This study aimed to investigate the potential protective effects of diminazene, an activator of angiotensin II converting enzyme (ACE2), on kidney function and structure in rats with acute kidney injury (AKI) induced by the anticancer drug doxorubicin (DOX). The impact of diminazene was compared to that of two other drugs: the ACE inhibitor lisinopril and the angiotensin II type 1 (AT1) receptor blocker valsartan. Rats were subjected to a single intraperitoneal injection of DOX (13.5 mg/kg) on the 5th day, either alone or in combination with diminazene (15 mg/kg/day), lisinopril (10 mg/kg/day), or valsartan (30 mg/kg/day) for 8 consecutive days. Various markers related to kidney function, oxidative stress, and inflammation were measured in plasma and urine. Additionally, kidney tissues were assessed histopathologically. DOX-induced nephrotoxicity was confirmed by elevated levels of plasma urea, creatinine, and neutrophil gelatinase-associated lipocalin (NGAL). DOX also led to increased urinary N-acetyl-β-D-glucosaminidase (NAG) activity and decreased creatinine clearance, albumin levels, and osmolality. Moreover, DOX caused a reduction in renal oxidative stress markers, including superoxide dismutase (SOD), glutathione reductase (GR), and catalase activities, while increasing malondialdehyde (MDA) levels. It also raised plasma inflammatory markers, tumor necrosis factor alpha (TNF-α) and interleukin 1 beta (IL-1β). Concurrently administering diminazene significantly mitigated these DOX-induced changes, including histopathological alterations like renal tubule necrosis, tubular casts, shrunken glomeruli, and increased renal fibrosis. Similar protective effects were observed with lisinopril and valsartan. These protective effects, at least in part, appear to result from the anti-inflammatory and antioxidant properties of these drugs. In summary, this study suggests that the administration of diminazene, lisinopril, or valsartan had comparable effects in ameliorating the biochemical and histopathological aspects of DOX-induced acute kidney injury in rats.

## Highlights

• Doxorubicin induces AKI by formation of reactive oxygen species and activation of pro-inflammatory pathways.
• Diminazine, lisinopril and valsartan have comparable protective effects on AKI caused by doxorubicin.
• Diminiazine, lisinopril, and valsartan exhibit salutary effects, partly due to anti-inflammatory and antioxidant properties.

## Graphical Abstract

**Figure:**
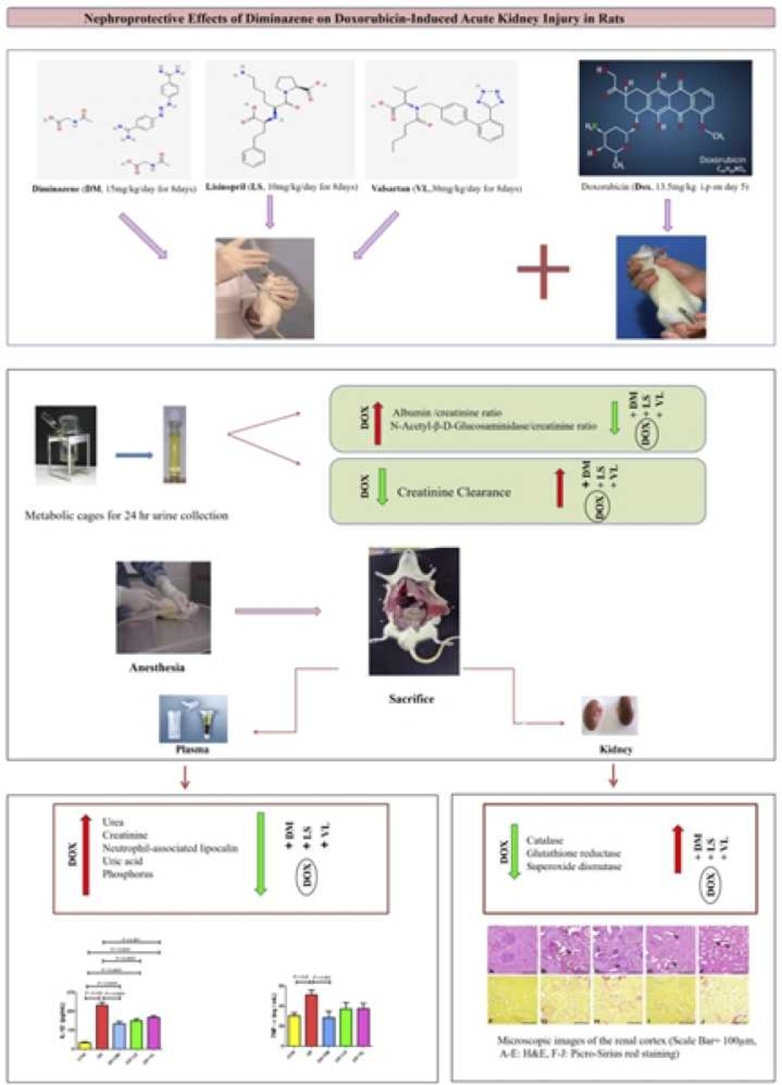

## 1 Introduction

Doxorubicin (DOX) is an effective antineoplastic agent widely used in the treatment of various hematological and solid tumors. However, its clinical utility is complicated by its toxic effects on multiple organs and tissues, notably the heart and kidneys [1]. DOX tends to accumulate in the glomerulus, leading to significant nephropathy that is characterized by glomerular and tubular atrophy, podocyte injury and increased glomerular capillary permeability [1]. DOX-iron complexes promote the release of reactive oxygen species (ROS) and perturb the mitochondrial electron transport chain [2]. In addition to oxidative stress, accumulating evidence suggests that the pathogenesis of DOX toxicity is mediated by inflammation, apoptosis and pyroptosis [3], [4], [5]. The current approach for preventing chemotherapy-induced nephrotoxicity is suboptimal, involving regular monitoring of renal function, increased hydration, dose adjustments and drug cessation [6]. Therefore, further studies are needed to develop protective measures and novel therapeutic strategies against the adverse effects of DOX therapy.

Drugs targeting the renin-angiotensin system (RAS), such as angiotensin II type 1 receptor (AT_1_R) blockers, angiotensin converting enzyme (ACE) and renin inhibitors, have shown positive outcomes in preserving renal structure and function against DOX-induced renal toxicity in numerous experimental studies [7], [8], [9], [10]. The mechanisms underlying these renoprotective effects of RAS inhibition are multifactorial, complex and not fully understood. However, they include a combination of antioxidative stress, anti-inflammatory, antiapoptotic, antiproliferative and antifibrotic actions [8], [9], [10]. Furthermore, while these drugs offset the pathological consequences of conventional RAS activation, they simultaneously shift the balance towards the nonconventional, protective axis of RAS, mediated by ACE2, Ang1–7 and angiotensin II type 2 receptor (AT2R) [8]. In keeping with this, ACE2 activators and Ang1–7 analogues have emerged in recent years with the pharmacological potential to improve renal function and structure in various experimental kidney disease models [11], [12], [13].

In a previous study, we reported the therapeutic potential of diminazene, an ACE2 activator, in an experimental rat model of chronic kidney failure [14]. Diminazene is a phenylhydrazine originally used as a chemotherapeutic agent for trypanosomiasis in domestic animals. In recent years, its therapeutic role has been demonstrated in various animal models with renal dysfunction including gentamicin-induced acute renal injury [15], renal ischemia/reperfusion [16], type 1 diabetes [17] and subtotal nephrectomy [18]. These findings support the renoprotective actions of diminazene, however its role in preventing DOX-induced AKI remains unknown.

Lisinopril is a non-sulfhydryl ACE inhibitor that is widely prescribed for the management of hypertension, heart failure and diabetic nephropathy. Lisinopril dilates renal blood vessels and reduces intraglomerular pressure, ultimately preserving renal function and ameliorating proteinuria and glomerular injury in various renal disorders [19], [20]. In addition, ACE inhibitors are known to possess antioxidant and free radical scavenging properties and have therefore been extensively explored for their potential role in alleviating DOX-induced organ toxicities [21], [22], [23]. In a recent study, lisinopril was shown to improve renal function and reduce free radical damage and apoptosis in DOX-treated AKI in rats [9]. On the other hand, valsartan is a selective AT_1_R blocker also commonly used as an antihypertensive agent and in the treatment of heart failure. Numerous studies indicate that valsartan produces a range of antioxidant, anti-inflammatory and antiapoptotic effects [7], [24]. Similar to lisinopril, several studies demonstrated the protective effects of valsartan against DOX-induced organ injuries, both in the clinical setting and in experimental animal models [7], [24], [25]. For example, treatment with valsartan in DOX-induced rats significantly improved renal structure, function, and oxidative stress markers [7].

Therefore, we aimed to investigate whether diminazene can protect kidney function and structure in AKI caused by DOX and examined the mechanisms underlying this effect. This experimental animal model closely mirrors the human pathophysiological features of AKI and has been extensively used to study the mechanisms underlying AKI, as well as to test pharmacological agents in managing chemotherapy-induced nephrotoxicity [9], [26], [27]. Finally, we compared the effect of diminazene treatment with lisinopril and valsartan.

## 2 Materials and methods

### 2.1 Drugs and chemicals

Diminazene aceturate, was bought from Abcam (Cambridge, UK). Lisinopril manufactured by AstraZeneca Pharmaceuticals Co. Ltd (Cheshire, UK) and valsartan manufactured by Novartis Pharma (Basel, Switzerland) were purchased from Muscat Pharmacy. DOX manufactured by Fresenius Kabi Oncology Ltd. (Gurgaon, India) was purchased from Sultan Qaboos University Hospital Pharmacy. The remaining chemicals used in this study were of the highest available purity grade.

### 2.2 Animals

Male Wistar rats weighing between 200 and 250 g were collected from the Small Animal House at Sultan Qaboos University. These rats were accommodated in a controlled environment where the temperature was maintained at 22 ± 2 °C, the relative humidity was approximately 60%, and a 12-hour light-dark cycle was implemented. They were provided with a standard diet and access to tap water without restrictions.

Every aspect of our experimental plans received approval from the Medical Research Committee at the College of Medicine and Health Sciences, Sultan Qaboos University. Additionally, the University Animal Ethics Committee at Sultan Qaboos University sanctioned the experimental protocol (SQU/AEC/2019–20/4). All procedures related to the handling and care of the animals strictly adhered to both national and international laws and regulations.

### 2.3 Experimental design

Thirty male Wistar rats were randomly divided into five equal groups and received the following treatments over an 8-day period:

Group 1: Control Group - These rats were given a regular diet for 8 days and received a saline injection on the 5th day.

Group 2: DOX Group - Similar to Group 1, these rats received a regular diet for 8 days but were injected intraperitoneally with DOX at a dose of 13.5 mg/kg on the 5th day of treatment.

Group 3: DOX + Diminazene Group - These rats were orally administered diminazene (dissolved in distilled water at a daily dose of 15 mg/kg) for 8 days, with DOX administered on the 5th day of treatment, similar to Group 2.

Group 4: DOX + Lisinopril Group - Rats in this group received lisinopril (suspended in distilled water at a daily dose of 10 mg/kg) orally for 8 days, along with DOX on the 5th day, as in Group 2.

Group 5: DOX + Valsartan Group - These rats were treated with valsartan (suspended in distilled water at a daily dose of 30 mg/kg) along with DOX on the 5th day, following 8 days of treatment, mirroring Group 2.

The doses of diminazene, lisinopril or valsartan and adenine were chosen according to previous studies [28], [29], [30].

One day prior to euthanizing the rats, urine was collected from each rat over a 24-hour period, and its volume was measured. After the treatment period, the rats were anesthetized using a combination of ketamine (75 mg/kg) and xylazine (5 mg/kg) administered intraperitoneally. Blood was then collected from the abdominal aorta into heparinized tubes and centrifuged at 900 x g for 15 min at 4 °C to separate plasma, which was stored frozen at −80 °C for later biochemical analysis. The rats were euthanized with an overdose of anesthesia, and their kidneys were removed, washed with ice-cold saline, blotted with filter paper, and weighed. A small section of the right kidney was preserved in 10% buffered formalin for histological analysis. The remaining portions of both the right and left kidneys were individually wrapped in aluminum foil, dipped in liquid nitrogen, and stored at −80 °C for analysis within ten days or less.

### 2.4 Biochemical analysis

#### 2.4.1 Kidney function markers

Plasma levels of urea, creatinine, uric acid, calcium, phosphorus, and urine concentrations of albumin and creatinine were quantified using an autoanalyzer (Mindray BS-120 chemistry analyzer, Shenzhen, P.R. China), as previously outlined [31], [32]. Plasma levels of neutrophil gelatinase-associated lipocalin (NGAL) were quantified using ELISA kits (Thermo Fisher Scientific, Inc, Walthman, MA, USA). Urinary NAG activity was assessed by standard spectrophotometry methods using Biovision kit (Milpitas, CA, USA). Urine osmolality was determined using Osmomat 3000 osmometer (Gonotec GmbH, Berlin, Germany), which relies on freezing point depression. Creatinine clearance (mL/min) was computed using the following formula:

(Urinary creatinine in µmol/L) × (Urine volume in mL/24 hr) / (Plasma creatinine in µmol/L) × 1440·

#### 2.4.2 Cytokines

Plasma levels of tumor necrosis factor alpha (TNF-α) and interleukin-1β (IL-1β) were quantified using ELISA kits from Thermo Fisher Scientific, Inc. (Walthman, MA, USA).

#### 2.4.3 Oxidative stress and antioxidant markers

For the assessment of kidney superoxide dismutase (SOD), catalase (CAT), glutathione reductase (GR) and malondialdehyde (MDA) levels, standard spectrophotometry methods using Biovision kit (Milpitas, CA, USA) were employed, as previously described [33], [34].

#### 2.4.4 Histopathological analysis

Renal tissue samples were extracted from the rats and preserved in 10% neutral buffered formalin before undergoing routine processing for histopathological evaluation. Sections of paraffin measuring 3–4 µm in diameter were prepared and subsequently stained with Hematoxylin and Eosin (H & E), as well as Picro-Sirius red (ab150681, Abcam). To evaluate renal tubular necrosis, a semi-quantitative scoring system was employed, as previously outlined by Ali et al. [33]. This system operates on a scale from 0 to 4, where 0 signifies normal tissue with no necrosis, 1 indicates less than 10% necrosis, 2 corresponds to 10–25% necrosis, 3 represents 26–75% necrosis, and 4 denotes over 75% necrosis. Three random microscopic fields at 40X magnification were observed in each kidney section from each rat across the five groups, and the mean percentage was transformed into the corresponding lesion score value. Fibrosis in kidney tissue samples was assessed through the utilization of the Picro-sirus red stain, which specifically colors collagen in red. Evaluation of the Sirius red-stained slides was conducted in accordance with the methodology outlined by Manni et al. [34]. An Olympus BX51 microscope equipped with an Olympus DP70 camera was employed for slide examination. Three random photomicrographs of the renal cortex were captured using a 40X objective lens. Images were acquired from each kidney of every rat within the five groups and stored as TIFF 24-bit RGB color image files. The camera and microscope settings remained consistent throughout the imaging process. Image analysis was performed utilizing the ImageJ® image analysis software (http://rsbweb.nih.gov/ij/). Photomicrographs were transformed into grayscale, and the red-stained collagen was isolated through the hue histogram filter, followed by the calculation of the separated area as a percentage. The fibrosis index percentage was determined to assess the collagen content in the tissues by computing the ratio of the mean area with Sirius red-stained positive collagen to the entire mean area of each photomicrograph for each rat.

### 2.5 Statistical analysis

The data was presented as mean values along with their standard error of the mean (SEM). Statistical analysis involved the use of one-way analysis of variance followed by Bonferroni's multiple comparison tests, conducted using GraphPad Prism version 5.03 from San Diego, CA, USA. A significance level of P < 0.05 was applied to determine statistical significance.

## 3 Results

### 3.1 Effect on physiological parameters

DOX significantly reduced body weight, urine output, urine osmolality, food intake and fecal output with no significant changes in the relative kidney or water intake (Table 1). Cotreatment with diminazene and lisinopril significantly reversed the changes in water intake and urine osmolality but not on other parameters. Cotreatment with valsartan did not have any significant effects on any of the measured parameters.

**Table 1:** Effect of diminazene (DM), lisinopril (LS), or valsartan (VL) treatment on some physiological parameters in rats with Doxorubicin (DOX)-induced acute kidney injury (AKI).

Parameters/ Treatment	Control	DOX	DOX + DM	DOX + LS	DOX + VL
**Base line body weight (g)**	223.8 ± 6.45	226.8 ± 12.58	220.5 ± 8.72	223.0 ± 10.22	221.2 ± 10.20
**Final body weight (g)**	255.8 ± 6.61	220.0 ± 12.71^a^	214.8 ± 8.17	218.3 ± 9.82	214.8 ± 7.31^a^
**Body weight change (%)**	14.4 ± 1.61	-3.0 ± 0.65^a^	-2.5 ± 0.73^a^	-2.1 ± 0.77^a^	-2.5 ± 2.08^a^
**Relative kidney weight (%)**	0.63 ± 0.01	0.73 ± 0.06	0.67 ± 0.02	0.72 ± 0.04	0.66 ± 0.02
**Water intake (mL)**	19.2 ± 1.57	23.2 ± 1.7	15.8 ± 3.51^b^	13.3 ± 1.99^b^	23.0 ± 1.93
**Urine output (mL)**	7.1 ± 0.71	12.7 ± 1.55^a^	9.7 ± 2.09	11.0 ± 2.59	10.2 ± 1.01
**Urine osmolality**	1890 ± 58.4	670 ± 44.7^a^	1237 ± 194.9^a^^,^^b^	974 ± 43.7^a^^,^^b^	892 ± 34.6^a^
**Food intake (g)**	13.9 ± 0.95	0.9 ± 0.1^a^	1.4 ± 0.21^a^	2.3 ± 0.75^a^	1.6 ± 0.26^a^
**Fecal output (g)**	4.02 ± 0.57	0.6 ± 0.07^a^	0.8 ± 0.15^a^	1.15 ± 0.32^a^	0.65 ± 0.07^a^

a Significant from Control

b Significant from DOX; n = 6

### 3.2 Effect on renal function and injury markers

DOX-treated rats had significantly higher plasma phosphorus, creatinine, urea and NGAL levels while their plasma calcium levels were significantly lower compared with the control group (Table 2). Diminazene, lisinopril and valsartan significantly reduced the DOX-induced changes in these markers, except for plasma calcium levels where the reduction was statistically significant only with diminazene.

**Table 2:** Effect of diminazene (DM), lisinopril (LS), or valsartan (VL) treatment on some plasma parameters in rats with doxorubicin (DOX) - induced acute kidney injury (AKI).

Parameters/ Treatment	Control	DOX	DOX + DM	DOX + LS	DOX + VL
**Urea (mmol/L)**	3.85 ± 0.34	18.11 ± 2.22^a^	5.99 ± 0.44^b^	11.69 ± 1.95^a^^**,**^^b^	12.55 ± 1.34^**a, b**^
**Creatinine (μmol/L)**	24.65 ± 1.37	50.20 ± 6.75^a^	30.91 ± 1.24^b^	42.75 ± 5.98^a^^**,**^^b^	37.55 ± 5.52^a^^**,**^^b^
**Phosphorus (mmol/L)**	0.68 ± 0.04	1.36 ± 0.07^a^	0.88 ± 0.04^a^^**,**^^b^	0.92 ± 0.05^**a,**^^b^	1.09 ± 0.08^**a,**^^b^
**Calcium (mmol/L)**	0.94 ± 0.06	0.39 ± 0.02^a^	0.58 ± 0.04^a^^**,**^^b^	0.49 ± 0.05^a^	0.40 ± 0.05^a^
**NGAL (ng/mL)**	25.61 ± 2.12	41.09 ± 3.98^a^	30.14 ± 2.08^b^	26.04 ± 2.56^b^	33.44 ± 1.86^b^

a Significant from Control

b Significant from DOX; n = 6

**Fig. 1:**
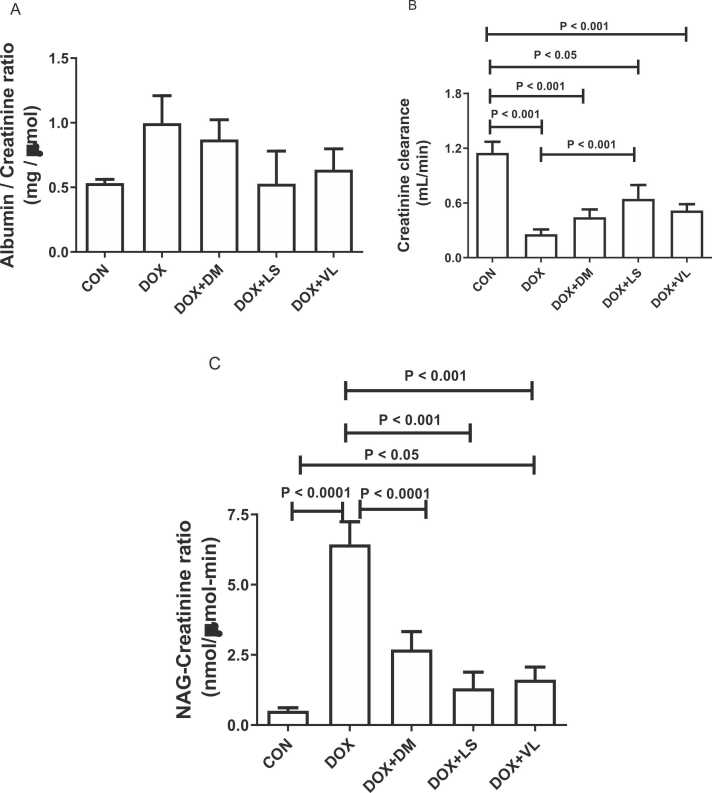
The effect of diminazene (DM, 15 mg/kg), lisinopril (LS, 10 mg/kg), and valsartan (VL, 30 mg/kg) on the following parameters in rats subjected to doxorubicin (DOX, 13.5 mg/kg) treatment: A. The urinary albumin/creatinine ratio B. Creatinine clearance C. Urinary N-Acetyl-β-D-Glucosaminidase (NAG)/creatinine ratio. DM, LS, and VL were administered orally to the rats throughout the experiment's duration. Acute Kidney Injury (AKI) was induced through a single intraperitoneal injection of DOX on the 6th day of the study. Urine samples were collected from the rats on the 9th day while they were placed in metabolic cages. The data provided represents the means with standard error of the mean (SEM) and is based on a sample size of six (n = 6).

Furthermore, DOX-treated rats had significantly higher NAG/creatinine ratio as well as decreased creatinine clearance compared with the control group (Fig. 1). Diminazene, lisinopril and valsartan significantly reduced the DOX-induced increases in NAG/creatinine ratio. Lisinopril also showed a significant improvement in creatinine clearance.

### 3.3 Effect on uric acid and inflammatory markers

**Fig. 2:**
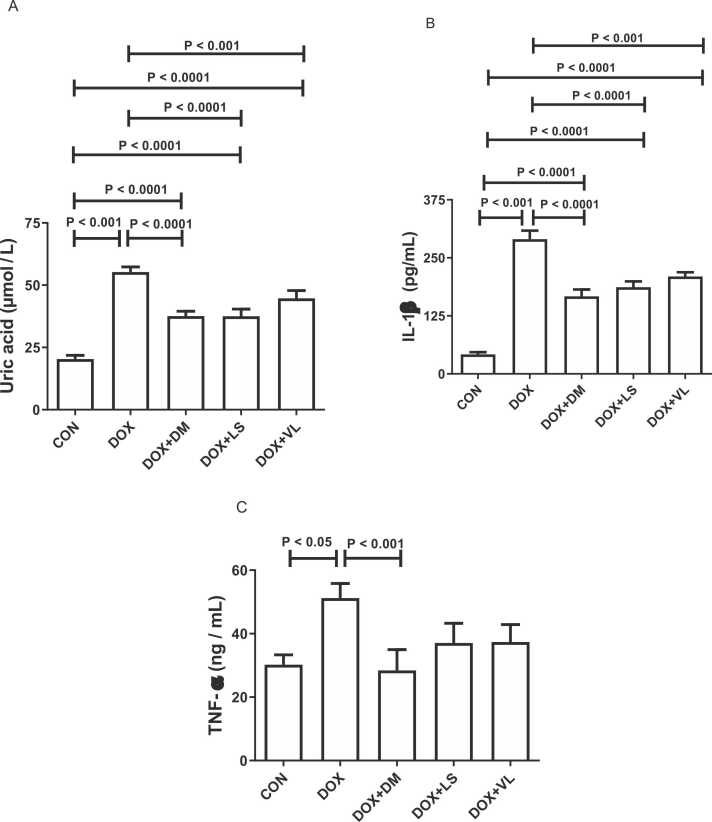
The effect of diminazene (DM, 15 mg/kg), lisinopril (LS, 10 mg/kg) and valsartan (VL, 30 mg/kg) on the levels of the following parameters in rats exposed to doxorubicin (DOX) treatment: A. Blood uric acid; B. Interleukin-1β (IL-1β); C. Tumor necrosis factor-alpha (TNF-α). Throughout the duration of the experiment, DM, LS, and VL were orally administered to the rats. Acute Kidney Injury (AKI) was induced by a single intraperitoneal injection of DOX at a dose of 13.5 mg/kg on the 6th day of the study. The data provided represents the means along with their standard error of the mean (SEM) based on a sample size of six (n = 6).

DOX significantly increased plasma uric acid, TNF-α and IL-1β (Fig. 2). Cotreatment with diminazene significantly reduced the DOX-induced changes in these parameters while lisinopril and valsartan significantly reduced plasma uric acid and IL-1β only.

### 3.4 Effect on oxidative stress markers

**Fig. 3:**
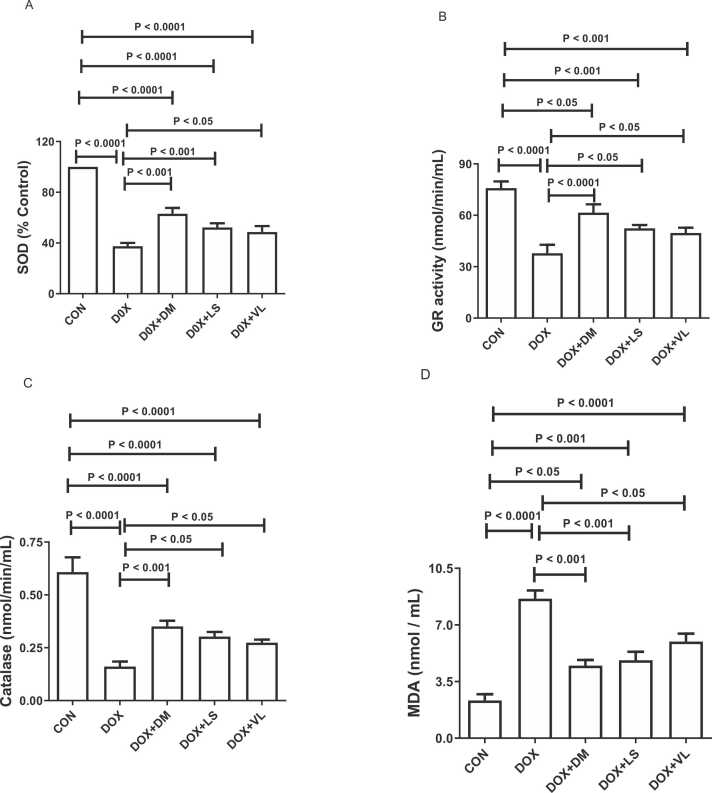
The effect of diminazene (DM, 15 mg/kg), lisinopril (LS, 10 mg/kg) and valsartan (VL, 30 mg/kg) on A. Renal superoxide dismutase (SOD); B. Glutathione reductase (GR); C. Catalase; and D. Malondialdhyde (MDA) in rats treated with doxorubicin (DOX). Throughout the experiment, DM, LS, and VL were given orally to rats. AKI was caused by a single intraperitoneal injection of DOX (13.5 mg/kg) on the 6th day of the experiment. The data provided represents the means along with their standard error of the mean (SEM) based on a sample size of six (n = 6).

DOX significantly decreased SOD, GR activity and catalase levels and increased MDA levels compared with the control group (Fig. 3). Cotreatment with diminazene, lisinopril or valsartan significantly attenuated the DOX-induced changes in these parameters.

### 3.5 Histopathology

**Fig. 4:**
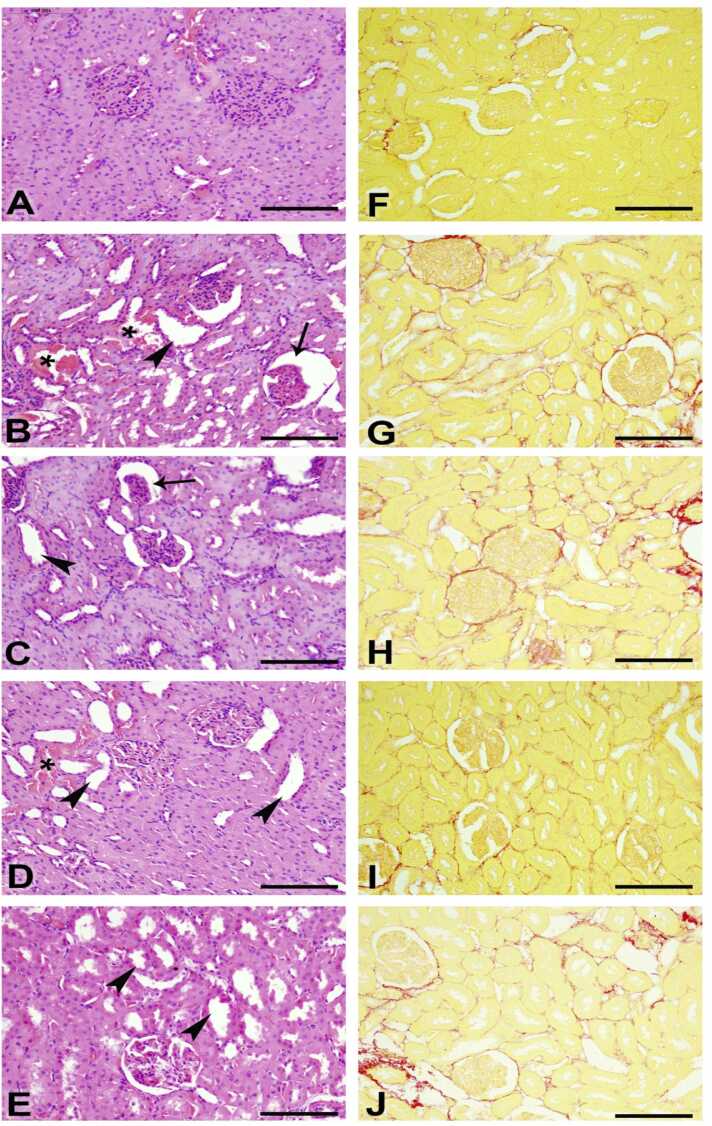
Microscopic images of the renal cortex (Scale Bar= 100 µm, A-E: H&E, F-J: Picro-Sirius red staining). (A) Control group displaying regular renal histology characterized by intact glomerular tufts and renal tubules (lesion score 0). (B) Doxorubicin (DOX) group showcasing pronounced cystic dilatation (marked by asterisk), basophilia, necrosis (indicated by arrow) of renal tubules, and the presence of tubular casts (arrowheads) along with shrunken glomeruli (lesion score 4). (C) DOX plus diminazene group presenting significant basophilia of renal tubules (arrow) along with the presence of tubular casts (arrowhead) and intact glomerular tufts (lesion score 2). (D) DOX plus lisinopril group demonstrating dilatation (asterisk) and necrosis (arrow) of a few renal tubules while maintaining intact glomeruli (lesion score 3). (E) DOX plus valsartan group displaying basophilia of renal tubules (arrows) with intact glomeruli (lesion score 2). (F-J) Depicting the distribution of collagen fibers stained in red and non-collagen structures stained in yellow in all five groups.

Examination of the renal tissues in rats from various groups under a microscope revealed different findings: In the control group (Fig. 4A), the renal histology appeared normal, with intact glomerular tuft and renal tubules (lesion score 0). The DOX-treated group (Fig. 4B) exhibited significant cystic dilatation, basophilia, and necrosis of renal tubules, along with tubular casts and shrunken glomeruli (lesion score 4). The DOX plus diminazene group (Fig. 4C) showed marked basophilia of renal tubules with the presence of tubular casts and intact glomerular tuft (lesion score 2). In the DOX plus lisinopril-treated group (Fig. 4D), renal tissues displayed dilatation and necrosis of a few renal tubules with intact glomeruli (lesion score 3). The kidney tissues from the DOX plus valsartan group (Fig. 4E) showed basophilia of renal tubules with intact glomeruli (lesion score 2).

In all five groups, the distribution of collagen fibers, stained in red, and non-collagen structures, stained in yellow, was demonstrated using Picro-sirus red stain (Fig. 4F-J), and the fibrosis index percentage was calculated and presented in Table 3.

**Table 3:** Effect of Diminazene (DM), Lisinopril (LS) or Valsartan (VL) treatment on histopathological assessment of kidney sections in rats with doxorubicin (DOX)-induced acute kidney injury (AKI).

Treatment/Assessment	Acute tubular necrosis		Fibrosis index %
	%	Lesion score	
Control	0.0 ± 0.0	0	4.22
**DOX**	76.67 ± 2.11^a^	4	23.43
DOX + DM	27.22 ± 3.15^a^^**,**^^b^	3	19.74
DOX + LS	21.12 ± 3.91^a^^**,**^^b^	2	10.76
DOX + VL	37.23 ± 1.59^a^^**,**^^b^	3	18.34

a Significant from Control

b Significant from DOX; n = 6

## 4 Discussion

We examined the effect of diminazene (13.5 mg/Kg) on AKI model and compared it to lisinopril and valsartan. AKI in our animal models was characterized by a reduction in body weight, urine output, urine osmolality, food intake and fecal output without significant changes in renal morphometry. These changes were accompanied by impaired renal function, elevated renal injury biomarkers, decreased antioxidant activity and increased signals of renal oxidative stress and proinflammatory cytokines. Histological findings showed extensive glomerular and tubular injury. Our results are consistent with clinical and experimental studies that have demonstrated DOX-induced multi-organ injury [4], [35], [36], [37].

Diminazine is a widely used antiparasitic drug which potently induces ACE2 as an off-target effect [38]. The main biological function of ACE2 is to counteract the pathophysiological actions of ACE by converting Ang II into Ang 1–7 which acts on the Mas oncogene receptor (MasR) [39]. The ACE2/Ang 1–7/MasR axis has multiple renoprotective actions. For example, it increases sodium excretion, reduces renal oxidative stress and inflammation and inhibits tissue fibrosis and remodeling [40], [41]. In this study, cotreatment with diminazene did not reverse the DOX-induced changes in body weight, urine output or food intake. However, it significantly improved most of the renal function indices, including attenuated urine osmolality, phosphorus, urea and creatinine levels. In terms of renal injury markers, the only improvement observed with diminazene treatment was in relation to the NAG/creatinine ratio. Corresponding with these results, substantial improvements were detected in the histopathological tubular injury, tubular necrosis and renal fibrosis induced by DOX treatment. In this respect, the effect of diminazene was similar to that of lisinopril and valsartan. However, the glomerular damage induced by DOX was not completely reversed by diminazene treatment, which likely explains its failure to correct markers of glomerular function, such as the albumin/creatinine ratio and creatinine clearance. Indeed, DOX tends to accumulate in the glomerulus, leading to significant damage to its and compromising glomerular filtration barrier [7], [42].

We further examined different oxidative and inflammatory markers to establish the possible mechanisms underlying the renoprotective actions of diminazene in DOX-induced AKI. Oxidative stress is hypothesized to be a key driver of DOX-induced nephrotoxicity [4]. Exposure to DOX increases superoxide levels, induces lipid peroxidation and reduces natural antioxidant compounds and free radical scavengers [2], [3]. In our study, DOX-treatment increased MDA levels, decreased SOD levels and reduced the enzyme activity of catalase and glutathione reductase, thus supporting the role of oxidative stress in DOX-induced nephrotoxicity. These changes were significantly reduced with diminazene treatment and were comparable to the reductions seen with lisinopril and valsartan treatment. The current results corroborate our earlier work demonstrating improved antioxidant indices in adenine-induced CKD rats treated with diminazene [14]. Diminazene has also been shown to reduce oxidative stress in animal models of renal ischemia/reperfusion injury and renal radiation-induced injury, as well as in various other cells and organs, including the liver, the endothelium and erythrocytes [16], [42], [43], [44], [45]. The antioxidant effect of diminazene may be explained by the actions of Ang1–7 subsequent to ACE2 activation [39]. Recent studies also indicate that diminazene reduces ROS generation by downregulating the expression of NAPDH oxidase independently of ACE2 activation [42]. Contrary to these findings, administration of diminazene at therapeutic doses was shown to promote oxidative stress in healthy male Wistar rats and female mice models of CKD [46], [47]. Treatment with standard doses of diminazene has also been linked to nephrotoxicity in domestic livestock, potentially due to the persistence of its metabolites in the kidneys for several weeks [46]. Therefore, further studies are needed to establish the exact pharmacological mechanisms and potential toxicity of diminazene in the kidney.

DOX administration significantly increased plasma uric acid and the proinflammatory cytokines TNF-α and IL-1β, supporting previous evidence of DOX-induced alterations of inflammatory markers [3], [4]. Hyperuricemia alters the cellular, morphological and functional profiles of the kidney through various mechanisms. Uric acid is recognized by various Nod-like and toll-like receptors, eliciting a potent inflammatory cascade which includes the release and maturation of IL-1β, as well as the up-regulation of TNF-α and TGF- β1 [48], [49]. Uric acid also activates the renin angiotensin aldosterone system, induces vascular smooth muscle cell proliferation, stimulates oxidative stress, promotes cellular apoptosis and enhances the production of extracellular matrix proteins, collectively contributing to glomerulosclerosis and renal fibrosis [48], [49]. In our study, uric acid and IL-1β were ameliorated by diminazene, lisinopril and valsartan while only diminazene attenuated TNF-α, suggesting a superior anti-inflammatory action. Our results align with several other studies supporting the protective association of diminazene against immune system perturbations in various experimental animal models [42], [50], [51]. Indeed, diminazene is known to inhibit a number of intracellular molecules and transcription factors that regulate cytokine and chemokine production [52], [53]. While it is largely speculated that the anti-inflammatory action of diminazene is mediated via the ACE2/Ang1–7/Mas axis, Rajapaksha et al. [42] have shown that diminazene reduces liver TNF-α expression and TNF-α secretion by Kupffer cells independently of ACE2 activation. Recent in vitro studies also indicate that the suppression of mitochondrial ROS generation by diminazene directly contributes to its anti-inflammatory action [44]. Taken together, our study suggests that the renoprotective effect of diminazene in DOX-induced AKI occurs, at least in part, through anti-oxidative stress and anti-inflammatory mechanisms. In this study, plasma or tissues ACE2, angiotensin (1−7) or angiotensin II levels were not measured. Hence, the protective effect of diminazene could have a component that is not dependent on activation of ACE2.

## 5 Conclusions

Treatment with diminazene at a dose of 15 mg/kg per day significantly reduced DOX-induced AKI nephrotoxicity and improved indices of renal structure and function. We showed that the renoprotective role of diminazene may be mediated by improvements in oxidative stress and inflammation.

## Ethical approval

The study was conducted in accordance with the Declaration of Helsinki, and approved by the Sultan Qaboos University Medical Research Ethics Committee (SQU/AEC/2019–20/4).

## Funding

This work was supported by a grant from 10.13039/501100004351Sultan Qaboos University (IG/MED//PHAR/21/02).

## Author statement

All the author declare that the work described has not been published previously, that it is not under consideration for publication elsewhere, that its publication is approved by all authors and tacitly or explicitly by the responsible authorities where the work was carried out, and that, if accepted, it will not be published elsewhere in the same form, in English or in any other language, including electronically without the written consent of the copyright-holder.

## CRediT authorship contribution statement

Conceptualization, **Yousuf Al Suleimani**, **Badreldin H Ali**, **Haytham Ali** and **Aly Abdelrahman**; Data curation, **Yousuf Al Suleimani**, **Badreldin H Ali** and **Aly Abdelrahman**; Formal analysis, **Yousuf Al Suleimani**, **Priyadarsini Manoj** and **Aly Abdelrahman**; Funding acquisition, **Yousuf Al Suleimani**; Investigation, **Haytham Ali**, **Priyadarsini Manoj** and **Ali Al Khamyas**; Methodology, **Yousuf Al Suleimani**, **Badreldin H Ali**, **Haytham Ali** and **Aly Abdelrahman**; Project administration, **Yousuf Al Suleimani** and **Aly Abdelrahman**; Validation, **Yousuf Al Suleimani**, **Badreldin H Ali** and **Aly Abdelrahman**; Writing – original draft, **Yousuf Al Suleimani** and **Raya Al Maskari**; Writing – review & editing, **Yousuf Al Suleimani**, **Badreldin H Ali** and **Aly Abdelrahman**. All the authors have read and agreed to the published version of the manuscript.

## Declaration of Competing Interest

The authors declare that they have no known competing financial interests or personal relationships that could have appeared to influence the work reported in this paper.

## Data Availability

Data will be made available on request.
